# Chemical characteristics of *Salix psammophila* sand barriers are accelerated degradation by ultraviolet irradiation and water

**DOI:** 10.3389/fpls.2024.1470347

**Published:** 2024-10-07

**Authors:** Ruidong Wang, Zhongju Meng, Yong Gao

**Affiliations:** College of Desert Control Science and Engineering, Inner Mongolia Agricultural University, Hohhot, China

**Keywords:** desertification control, *Salix psammophila* sand barriers, chemical characteristics, ultraviolet irradiation, biological resources utilization

## Abstract

The implementation of *Salix psammophila* sand barriers measures constitutes a crucial element in desertification control, providing a solid theoretical foundation for the future application and pretreatment of sand barriers in production practices. To address the specific damage types predominant in desert environments, we executed simulations of ultraviolet irradiation and rainfall phenomena on mechanical sand barriers in sandy areas and also inspected the variations in chemical properties during accelerated aging processes. The findings unequivocally demonstrate that: (1) The synergistic impact of ultraviolet irradiation and water accelerated the deformation and fracturing of the *S.psammophila* sand barriers, thereby causing a partial degradation of its chemical properties and conspicuous lignin oxidation; (2) The fissure of the sand barrier deepened, resulting in structural alterations. The existence of water expedites the degradation process of *S.psammophila* sand barriers under ultraviolet irradiation. (3)With respect to the binding form of C atoms, the carbon atoms at *S.psammophila* sand barriers were highly oxidized after 576 hours of accelerated aging. The components of C2 (C-O) and C3 (C=O) rising to 40.16% and 12.24% respectively, while the components of C1 (C-C) declined to 47.60%. The amount of hydroxyl (O-C-O), carbonyl (C=O), and carboxyl (O-C=O) groups increases in line with the expansion of the contact area between the sand barrier structure and ultraviolet irradiation as well as water. More free radical substances are generated, thereby causing the chemical binding properties to tend to be more stable. In summary, Ultraviolet irradiation and water change are the primary factors influencing the degradation of *S.psammophil*a sand barriers material structure and properties. In future desertification control, it is imperative to focus on enhancing the longevity of sand barriers by ensuring their waterproofing capabilities and resistance to ultraviolet irradiation.

## 1 Introduction

The *Salix psammophila* sand barriers, functioning as a mechanical measure, exert a pivotal role in the engineering technology of desertification control and constitute an essential prerequisite for vegetation-based desertification control (Li and Ha, 2020; Gao et al., 2013). *S.psammophila* sand barriers are capable of enhancing the soil nutrient content and reducing the levels of fine particulate matter in the surface soil of arid areas (Hai et al., 2016; Gao and Yu, 1996). The application of *S.psammophila* sand barriers effectively mitigates the movement of sand dunes and accelerates vegetation recovery, making it extensively employed in desert regions characterized by frequent sand displacement in China (Gong, 2012). Based on comprehensive analysis and extensive research, it is apparent that the utilization of *S. psammophila* as a sand barrier in desertification prevention and control demonstrates significant long-term wind and sand fixation benefits when implemented in sandy areas (Wang et al., 2019; Wang et al., 2021a). However, in the atmospheric environment of the desert area, the tissues at both ends of the mechanical sand barrier are exposed, resulting in the termination of plant nutrient supply and subsequent cell tissue death. Moreover, various environmental and climatic factors inherent in nature trigger its degradation (Gao et al., 2013, Zhou et al., 2008). The degradation of *S.psammophila* sand barriers is accompanied by the occurrence of textural roughness, cracking, warping deformation, lodging, and other phenomena. This results in a certain degree of deterioration of the sand barriers and subsequently influences its durability of the wind and sand consolidation function (Gong, 2012; Rozema et al., 1997).

The degradation of the *S. psammophila* sand barriers is ascribed to various factors in the natural environment. The deterioration of its chemical constituents subsequently gives rise to the decline in its physical and mechanical properties, significantly influencing its utility value. Kocaefe et al. (2013) manifested that the degradation of lignin significantly affects the alteration of color and wettability on wood surfaces, with substantial degradation taking place during weathering subsequent to water treatment. Yamauchi et al. (2004) contends that the impact of ultraviolet radiation and water on the chemical composition of wood surpasses that of ultraviolet light alone. The *Albizia julibrissin* and *S.psammophila* were subjected to natural and artificial accelerated aging conditions, including UV exposure and water immersion, The findings revealed that both types of wood experienced lignin oxidation under varying weathering conditions, with a significant decrease in hemicellulose and lignin content after water weathering (Sudiyani, 1999). Owen et al. (1993) conducted light, rain, and combined rain and light tests on samples of pine and birch, leading to rapid changes in functional groups on the wood surface within a few hours. The chemical composition of wood underwent the most significant changes under the combined treatment of rain and light. The degradation of *S. psammophila* sand barriers weakens and loses its windbreak and sand fixation benefits, thus becoming one of the primary factors limiting the longevity of this protective structure (Wang et al., 2022). In conclusion, the protective properties of wood materials are affected by ultraviolet irradiation and moisture. However, the researchers mainly concentrated on enhancing water treatment based on environmental light to conduct artificial accelerated aging experiments. These were mainly aimed at comparing variations among different tree species or regions, with limited exploration into the deterioration mechanism of *S. psammophila* sand barriers under combined exposure to ultraviolet irradiation and water. Research potentially enables structural and performance modifications. Limited studies have been carried out on the physical and mechanical properties, chemical composition, chemical groups, and structure of wood materials. Furthermore, there is a dearth of real-time tracking observations and research experiments. This lack of knowledge impedes researchers from comprehending the degradation of *S.psammophila* sand barriers, particularly in deserts with fragile ecological environments.

When exploring the ecological protection benefits of *S.psammophila* sand barriers, it is indispensable to take into account the sustainable utilization of their resources. The degradation process of *S.psammophila* sand barriers exposed to atmospheric conditions is protracted, and controlling the combined influence of light and rainfall presents challenges. To simulate the failure mechanism of the sand barrier under the combined effect of ultraviolet irradiation and water (representing rainfall), we simulated the influence of ultraviolet irradiation and rainfall on the performance of *S.psammophila* sand barriers indoors.

## 2 Materials and methods

### 2.1 *Salix psammophila* sand barrier sample selection and experimental design

The *S.psammophila* sand barriers materials were collected from Hangjin Banner, Ordos, Inner Mongolia, China. Representative 3-5-year mature and healthy growing fresh *S. psammophila* branches were randomly selected in the collection area. S. psammophila branches with a diameter class of 7–13 mm were selected 20 cm from the ground as experimental materials. In accordance with the national forestry industry standard LY/T 2369-2014 “Testing Method for Physical and Mechanical properties of desert shrubs”, sawing and cutting were conducted (LY/T 2369, 2014). When selecting specimens, defective specimens formed during the sawing process, with overly rough surfaces, uneven thicknesses, no large number or obvious joints, cracks and other defects, were initially removed. The aim is to avoid the disparity in results caused by the varying physical, mechanical, and chemical properties and structures of *S. psammophila* at different locations, and to minimize the discreteness of the test results.

### 2.2 Experimental design of accelerated aging process

The accelerated aging test approach of the sand barrier was implemented in accordance with the national standard (ASTM154-06), and the artificial intense ultraviolet radiation environment box (AIUREB) was utilized to simulate the artificial accelerated ultraviolet irradiation. The total effective duration of outdoor ultraviolet irradiation is converted into the indoor simulation duration of ultraviolet irradiation. The reference standard of outdoor ultraviolet irradiation quantity is the natural condition of Kubuqi Desert area in Inner Mongolia,China. The average total solar ultraviolet irradiation ranged from 4860~6931 MJ/m^2^ during the period from 1961 to 2017. Affected by geographical location and climatic factors, the sunshine duration was relatively long. The annual total ultraviolet irradiation in the study area was 6581.2MJ/m^2^ (658.12 KJ/cm^2^). Among them, the total ultraviolet radiation accounted for 7% of the total solar radiation, that is, 46.07 KJ/cm². According to the irradiance meter, the ultraviolet irradiation intensity of the *S. psammophila* sand barriers sample is the greatest at a distance of 30 cm from the light source, and the average UV irradiation intensity is 0.024 W/cm².

Based on the conversion approach of the total effective duration of outdoor UV irradiation and that of indoor ultraviolet irradiation (Qi, 2011; Yan et al., 2001). We calculated the amount of ultraviolet irradiation in the pre-test to maximize the limit of test conditions and simulate the maximum degree of degeneration damage to the *S.psammophila* sand barrier under outdoor natural irradiation. During the experiment, the daily exposure time was controlled from 6:30 am to 22:30 every day. The total ultraviolet irradiation lasted for 16 h, with an interval of 8 h, to simulate the phenomenon of day and night alternation. Intermittent ultraviolet irradiation facilitates the cracking of the test material of *S. psammophila* sand barriers, thereby enabling its inner structure to receive ultraviolet radiation more rapidly (Ye and Huang, 2005). Among them, within a daily irradiation period of 16 hours, at a temperature of 40 ± 3°C and in an environment with 50% relative humidity, distilled water was intermittently sprayed for 3 hours to simulate the rain phenomenon. This intermittent spraying contributes to accelerating the aging process of the sand willow mechanical sand barrier test material. The ultraviolet irradiation aging experiment was carried out, resulting in an extended humidity aging time of 576 h. By performing calculations, a comparison between the combined treatment of light and humidity in this specific area and the simulated laboratory duration were obtained. The natural ultraviolet irradiation time, laboratory simulation time and radiation amount in the Kubuqi Desert area can be converted by the formula, as shown in **Table 1**.

**Table 1:** Accelerated aging process simulation time and irradiation amount of *S.psammophila* sand barrier.

Outdoor UV radiation time	Total outdoor radiation	Indoor UV radiation time	Total indoor radiation	Water spray
(y)	(KJ/cm^2^)	(d)	(KJ/cm^2^)	(h)
0	0	0	0	0
1	46.07	24	50.68	72
2	92.14	48	101.35	144
3	138.21	72	152.03	216
4	184.28	96	202.71	288
5	230.35	120	253.39	360
6	276.42	144	304.06	432
7	322.49	168	354.74	504
8	368.56	192	405.42	576

During the test, the temperature of the environmental chamber was controlled at 40 ± 3 °C to prevent the sample from undergoing thermal aging due to excessive temperature during ultraviolet aging, which could affect the accuracy of the test. After the test, the samples were respectively taken out to test the performance index of the *S. psammophila* sand barriers. After the conclusion of the test, the aging samples of each distinct ultraviolet irradiation treatment period were placed in an artificial climate chamber where the temperature was maintained at 20 ± 0.2°C and the relative humidity was 65% ± 5%. The moisture content of the samples was adjusted to conform to the range of 9% to 15% required by the national standard mechanical test. After the samples were dried and ground into powder, the main chemical functional groups and alterations in chemical bonds of various types of samples were tested. Meanwhile, samples were selected for sectioning to observe the structural changes.

### 2.3 Experimental test method

#### 2.3.1 Observation of the *Salix psammophila* sand barriers structure

Macroscopic structure observation. The samples retrieved from the field and the *S.psammophila* sand barriers treated in different rooms were polished, and the blocks were repaired with knives and other tools. Some samples were selected and observed with a stereomedical microscope (Leica SAPO, Leica, Switzerland).

Observation of microstructure: The parts of the sand barrier exposed to the atmosphere were selected in the field test, and some samples with significant changes and differences were chosen in the indoor simulation test for microstructure observation. Horizontal and radial test blocks were prepared in accordance with the national standard GB/T 29894-2013. After being vented in a water bath for 10 days, glacial acetic acid and 30% hydrogen peroxide in a ratio of 2:1 were used for softening for 30 minutes, it was washed with cold water several times to remove the acid, and soaked in ether solution for testing. Slides with a thickness of approximately 10 to 20 μm were prepared using a sliding microtome. (SM2400, Lycra, Germany), and then observed with a conventional light microscope and a scanning electron microscope.

#### 2.3.2 FTIR test analysis of *Salix psammophila* sand barriers

FTIR analysis of the samples of *S. psammophila* sand barriers was conducted using the traditional KBr tablet fabrication approach. The wood powder sample (2 mg) and potassium bromide (200 mg) of the *S. psammophila* sand barriers samples were evenly mixed and placed in the production mold, and round thin samples were fabricated under the conditions of 8 - 10 MPa for 2 minutes. The BRUKER (TENSON 27) infrared spectrometer was employed for the experiment. The spectrometer is equipped with a DTGS detector, and the infrared spectrum scanning range is 4000-400 cm^-1^, the resolution is 4 cm^-1^, scaned 32 times. The OPUS 6.5 analysis software was employed to undertake pretreatments such as baseline correction, atmosphere compensation, smoothing, and normalization respectively, for determining the relative intensity of the characteristic peaks of the main chemical components of *S. psammophila* sand barriers (Traoré et al., 2016; Boukir et al., 2019).

#### 2.3.3 Analysis of X-ray diffraction

X-ray diffraction analysis of the sand barrier samples was performed using an X-ray diffractometer (Philips X-Pert, Panalytical, Almelo, Netherlands) under Cu-Kα radiation (k = 0.154 nm). The test conditions were as follows: 2 s/step, 0.02 degree/step, 40 kV, and 30 mA, and the sample was spread within a range of 2θ = 5 to 40˚. The Cri (%) crystallinity index was described by the Segal method (Segal et al., 1959), using the following equation (Boukir et al., 2019).


(1) $$C\text{r}.I(\%)=\left[\left(I_{002}-I_{\text{am}}\right)/{\text{I}}_{002}\right]\times 100$$

where I_002_ is the diffraction peak intensity of the crystallization corresponding to the (002) plane at 2θ=22.5˚, and I_am_ is the diffraction intensity at 2θ=18˚.

#### 2.3.4 X-ray photoelectron spectroscopy

XPS analysis was performed using an X-ray photoelectron spectroscopy (Escalab 250Xi; Thermo Fisher Scientific, Waltham, Massachusetts, USA). In this study, *S. psammophila* sand barriers with different aging times (zero, five, and seven years) were selected, and small samples with dimensions of 10×10×2 mm (length × width × thickness) were cut. An aluminum target X-ray source with a monochromator (Al Kα, Hν=148671 eV) was used at 225 W (operating voltage 15 kV, emission current 15 mA). Furthermore, the pollution carbon (internal standard) was 284.8 eV, the minimum energy resolution was 0.48 eV Ag (3d5/2), and the minimum XPS analysis area was 15 μm. The data were processed using MDI.Jade 6.0 and Origin 2021 software.

## 3 Results

### 3.1 Damage phenomena of sand barriers during accelerated aging processes

**Figure 1:**
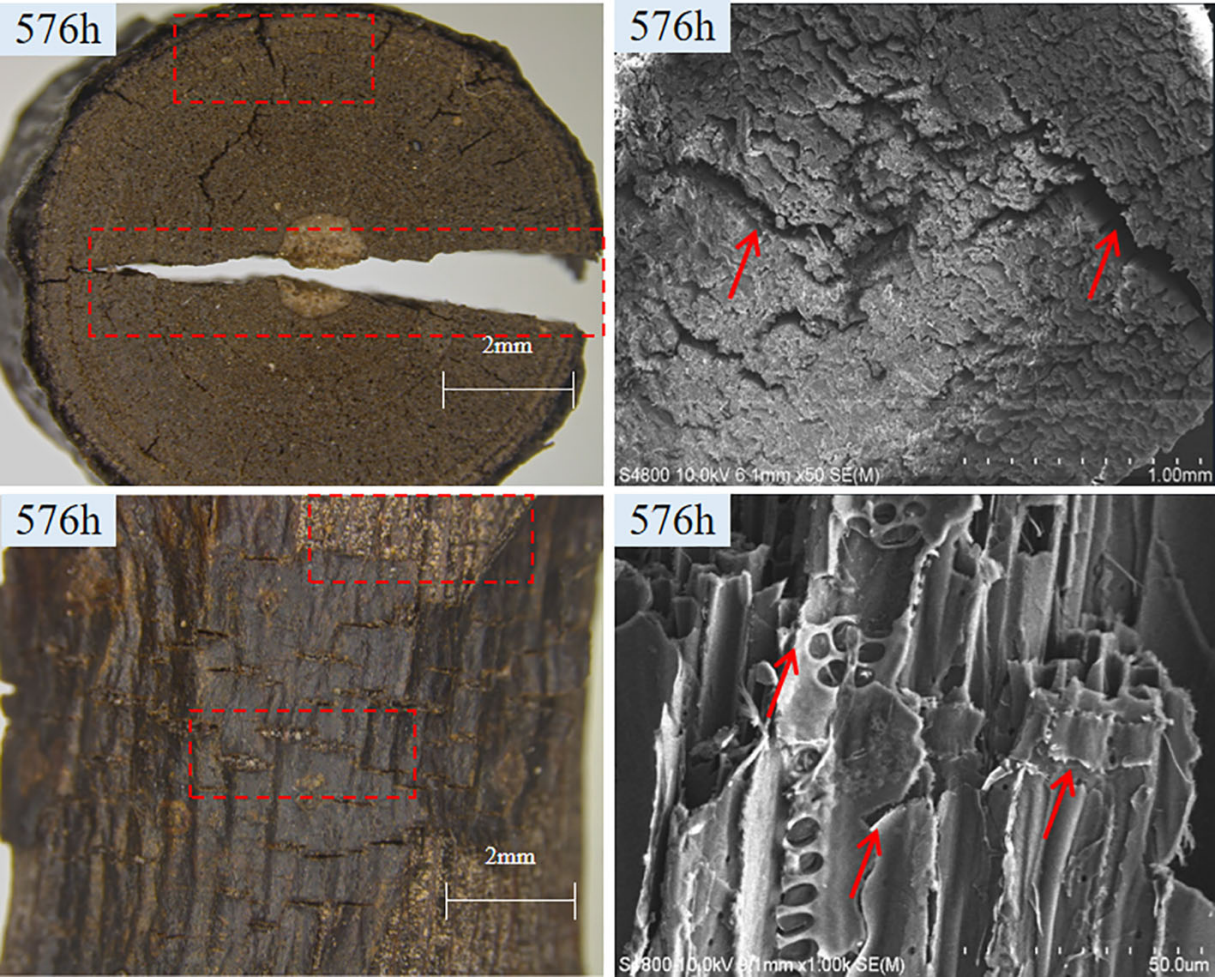
Macroscopic and microscopic structural alterations of *S.psammophila* sand barriers. (Red marks indicate points with obvious degradation characteristics).

As shown in **Figure 1**, after undergoing accelerated aging for 576 hours, notable changes were observed in the macrostructure and microstructure of *S.psammophila* sand barriers. The macroscopic structure exhibits a deepening and darkening of color, accompanied by a decrease or even disappearance of brightness and gloss. Additionally, there are prominent cracks present, with an increased number being observed, along with an evident shedding phenomenon on the epidermis. The microscopic structure reveals evident damage to the cell wall of *S.psammophila* sand barriers, characterized by conspicuous fractures, distorted and incomplete tracheid, as well as severe pore decomposition. Under the influence of water, the cell wall of *S.psammophila* sand barriers contracts inwardly, causing severe damage to the wood ray structure and the complete collapse of various tissues.

### 3.2 Chemical functional groups of sand barriers during accelerated aging processes

**Figure 2:**
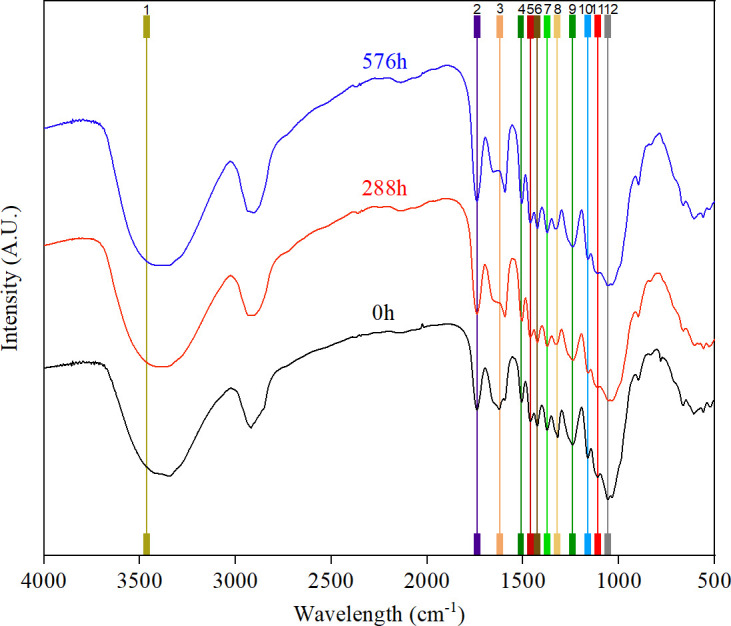
FTIR spectral characteristics of *S.psammophila* sand barriers.

As shown in **Figure 2**. In the wave number range of 4000~400 cm^-1^, the sand barriers exhibit primarily 12 distinctive absorption peaks. After accelerated aging for 288 h and 576 h, the predominant chemical functional groups of the sand barriers exhibited significant alterations. The wave peaks were observed at 1620 cm^-1^ (C=C group in the lignin skeleton), 1460 cm^-1^ (C-H group in lignin and carbohydrates), 1425 cm^-1^ (C-H group in lignin and hemicellulose), 1321 cm^-1^ (O-H group in lignin), and 1242 cm^-1^ (C-O group of guaiacyl unit and C-O group of lignin) exhibited a significant reduction in absorption peak intensity, indicating the occurrence of degradation within the lignin structure. The expansion vibration absorption at 1739 cm^-1^ (C=O groups in acetyl and carboxyl groups in hemicelluloses) was enhanced, leading to a significant increase in the content of carbonyl groups. The degradation of most organic matter existing in wood materials takes place due to exposure to sunlight, water, oxygen, and other environmental factors. The infrared spectrum reveal that the absorption peaks at 1373 cm^-1^ (C-H groups in cellulose and hemicellulose), 1161 cm^-1^ (C-O-C groups in cellulose and hemicellulose), and 1056 cm^-1^ (C-O groups in cellulose and hemicellulose) predominantly correspond to polysaccharides (cellulose and hemicellulose). Nevertheless, no substantial differences were witnessed, suggesting negligible overall alterations. In summary, considerable alterations were detected in the characteristic peaks of the chemical groups representing lignin throughout the accelerated aging process, resulting in the degradation of the macromolecular chemical constituents of lignin.Compared with lignin, the characteristic peaks representing cellulose and hemicellulose exhibited less variation, but they also played a role in the degradation of *S.psammophila* sand barriers.

### 3.3 X-ray diffraction and crystallinity of sand barriers during accelerated aging processes

**Figure 3:**
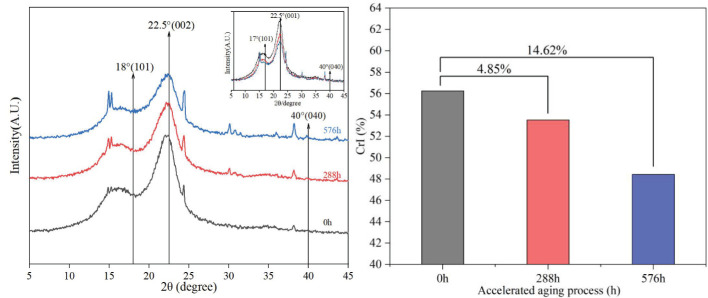
XRD characteristic diagram and crystallinity of *S.psammophila* sand barriers.

After accelerated aging for 288 h and 576 h, the XRD diffraction characteristics and crystallinity were depicted in **Figure 3**. After the accelerated aging treatment, no additional fiber beam diffraction peaks were witnessed, suggesting the maintenance of the intact lattice structure of cellulose. The crystallinity of *S.psammophila* sand barriers declined by 14.62%. This observation implies that the accelerated aging induced by ultraviolet irradiation and water can deteriorate lignin, cellulose, and hemicellulose within the sand barriers, resulting in the destruction of *S.psammophila* sand barriers cell wall and an accelerated deterioration process. Therefore, it can be deduced that the degradation of the scientific performance of the *S.psammophila* sand barrier in the atmospheric environment of sandy areas is ascribed to a combination of ultraviolet irradiation aging and water exposure, leading to the breakdown of the cellular structure and a significant deterioration of its protective capabilities.

### 3.4 X-ray photoelectron spectroscopy of sand barriers during accelerated aging processes

**Figure 4:**
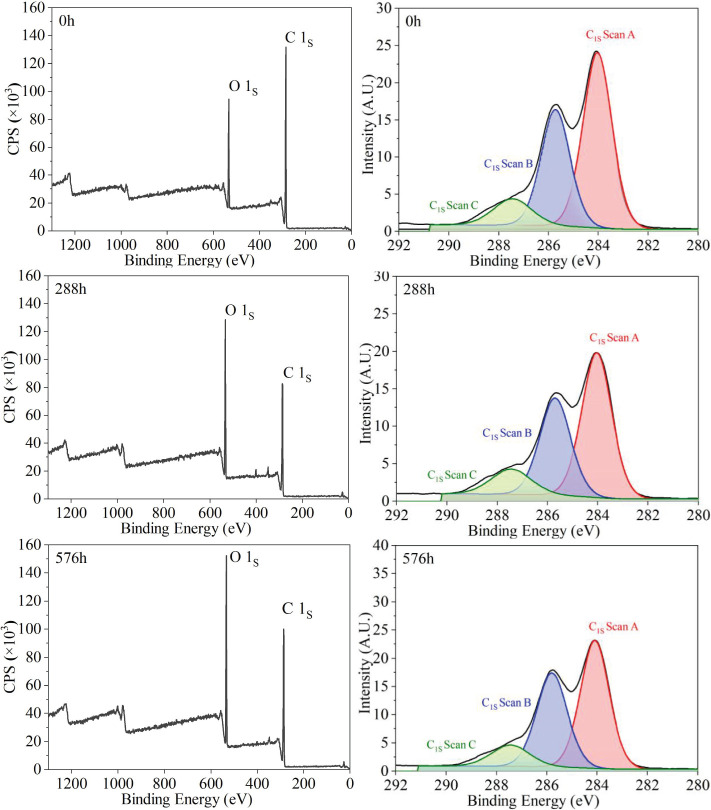
XPS wide scanning spectrum and C_1_s peak characteristic of *S.psammophila* sand barriers.

As shown in **Figure 4**. The XPS patterns after accelerated aging for 288 hours and 576 hours presented C1s peaks at an electron binding energy of approximately 285 eV, along with O1s peaks at an electron binding energy of 532 eV. The main chemical components of *S.psammophila* sand barrier are carbon, hydrogen, and oxygen elements. After accelerated aging for 288 h and 576 h, the elemental composition of the *S.psammophila* sand barriers is still dominated by carbon, hydrogen, and oxygen; however, their relative proportions have undergone changes.

The chemical information concerning the accelerated aging process of *S.psammophila* sand barriers is typically analyzed in accordance with the states of binding atoms of carbon and oxygen elements, as well as the O/C ratio. C1s peak fitting was carried out on the sand barrier during the accelerated aging process, leading to the identification of three distinct peaks, namely C1 (C-C), C2 (C-O), and C3 (C=O). Notably, their relative proportions manifested significant changes. The elemental composition was ascertained by calculating the proportion of each element based on the peak area in the wide scan map of *S.psammophila* sand barriers.

As shown in **Figure 4** and **Table 2**, The accelerated aging process of *S.psammophila* sand barriers manifested an ascending trend in the oxidation states C2 and C3, whereas a descending trend was witnessed in the content of C1. After accelerated aging for 288 hours, the O/C ratio increased from 0.23 to 0.40, while the content of C1 decreased from 73.01% to 52.07%. Additionally, C2 increased from 20.70% to 36.01%, and C3 rose from 6.29% to 11.94%. After 576 h of accelerated aging, the O/C ratio further elevated to 0.43, whereas the content of C1 decreased even more prominently to reach 47.60%. Furthermore, there was a considerable increase in C2 to approximately 40.16%, and an additional rise in C3 to approximately 12.24%. These findings imply that before the combined aging treatment, the main binding forms between carbon atoms were identified as mainly C-C and C-H interactions, followed by a certain presence of C-O bonds. In the hydroxyl (O-C-O), carbonyl (C=O), and carboxyl (O-C=O) configurations, element C exhibit a higher electron binding energy, indicating its continuous exposure to oxygen and water during accelerated aging. This leads to surface oxidation reactions that stabilize chemical bonds and result in enhanced binding properties. The O1 form represents the attachment of an oxygen atom as C=O, primarily originating from lignin with low binding energy due to double bond connections between carbon and oxygen. On the other hand, the O2 form arises from cellulose and hemicelluloses where oxygen is connected to carbon through single bonds, resulting in high binding energy.

**Table 2:** Accelerate the aging process types and binding energy positions of C_1_s peaks.

Element composition	Binding type	Binding energy/eV	Time/h		
			0h	288h	576h
C1/%	C-C, C-H	284.05	73.01	52.07	47.60
C2/%	C-O	285.75	20.70	36.01	40.16
C3/%	O-C-O, C=O	287.45	6.29	11.94	12.24
C1/C2	⎯⎯	⎯⎯	3.53	1.45	1.19
C_ox_/C_unox_	⎯⎯	⎯⎯	0.37	0.92	1.10
O1/%	O-C=O	532.25	97.90	96.80	98.08
O2/%	C-O-, C=O, C-O-C, O-C=O	530.25	2.10	3.20	1.92
O/C	⎯⎯	⎯⎯	0.23	0.40	0.43

**Figure 5:**
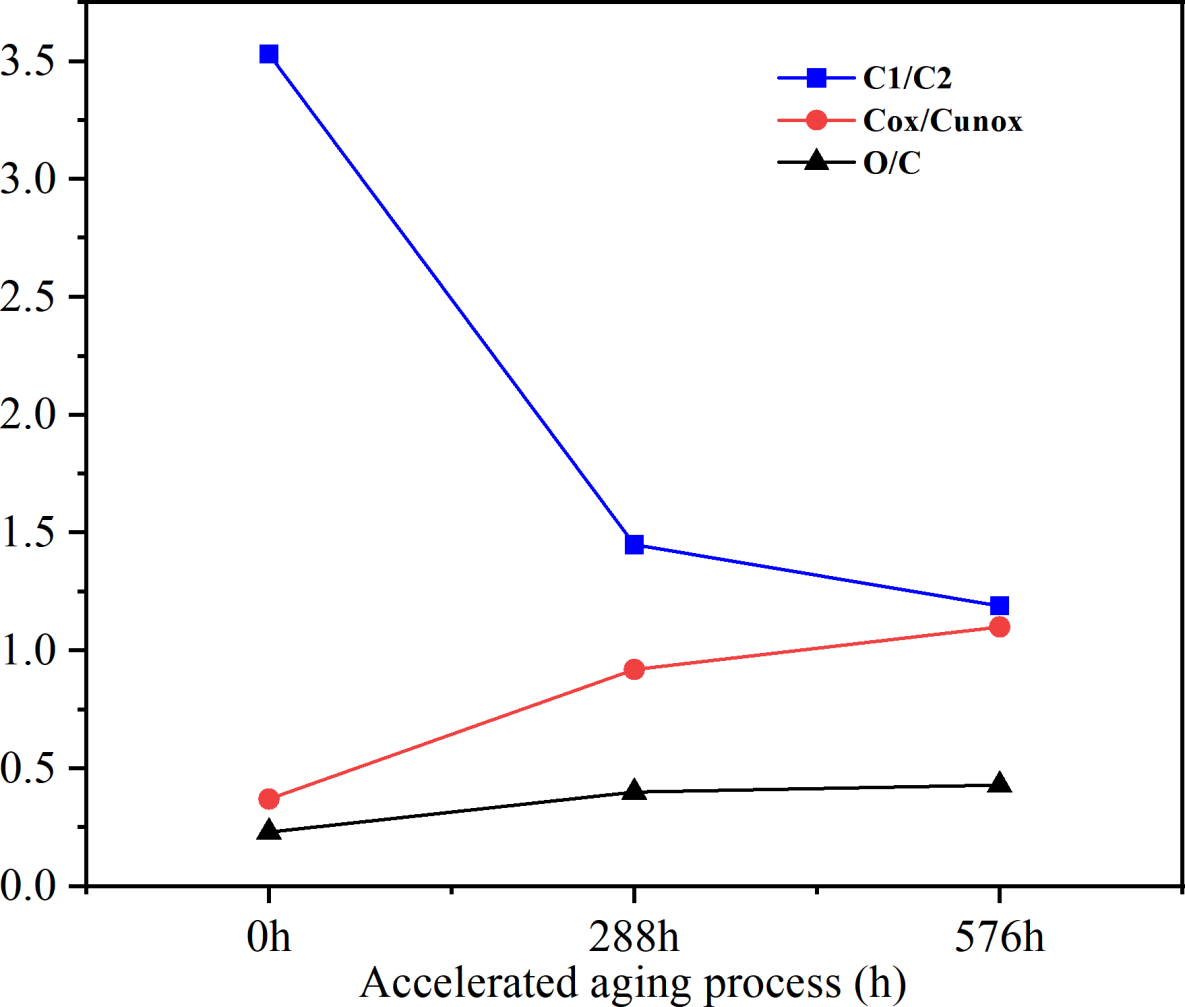
C1s and O/C changes of *S.psammophila* sand barriers.

As shown in **Figure 5**. The C1/C2 curve demonstrate a continuous decline in the ratio of C1 with increasing accelerate aging time, decrease from 3.53 to 1.45 after 288 h and further to 1.19 after 576 h. On the other hand, the Cox/Cunox curve reveal an increasing trend in the contents of C2 and C3, which rised from 0.37 to 1.10 after 576 h, indicating a rapid increase in hydroxyl, carbonyl, and carboxyl groups. The O/C ratio exhibited an increasing trend, indicating significant lignin degradation during the accelerated aging process. Moreover, under ultraviolet irradiation, the stability of lignin was found to be lower than that of polysaccharide, and notable changes were observed in the C1s component during the aging process accelerated by ultraviolet and water.

## 4 Discussion

The *S.psammophila* sand barriers manifested conspicuous damage, encompassing the emergence of penetrating cracks and substantial detachment of the surface, after being exposed to ultraviolet irradiation and accelerated aging by water for 288 hours and 576 hours. Wang et al. (2015) found that the prolonged exposure of wood to sunlight triggers a photochemical reaction, leading to the greying or darkening of the wood and subsequent decomposition. This process commences the formation of small dry cracks or fissures on the wood surface, which further extend upon contact with water. After 288 hours, a series of chemical reactions occurred on the surface of *S.psammophila* sand barriers. During this stage, the reaction rate is augmented and the degree of color change is more rapid, which is dependent on the treatment time and conditions (including water spray). These findings are consistent with previous research by Sudiyani et al. (2003). The intensification and structural alterations of the cracks imply that the synergistic effect of ultraviolet irradiation and water can accelerate the aging and deterioration process of *S.psammophila* sand barriers. According to Sudiyani et al. (2003), the water utilized during the artificially accelerated aging process seems to facilitate water infiltration into wood, thereby accelerating the degradation of wood (Sudiyani et al., 2003). Simultaneously, remarkable changes in the primary chemical properties of the *S.psammophila* sand barriers suggest its significant chemical reactivity under the combined effect of ultraviolet irradiation and water. This phenomenon can mainly be ascribed to the inherent wood composition of *S.psammophila* sand barriers, which facilitates the absorption of ultraviolet irradiation during aging processes and subsequently gives rise to the formation of free radical species. After the application of water, hydrogen peroxide is produced through the interaction of oxygen and water. The existence of free radicals and hydrogen peroxide initiates a series of chemical reactions, including molecular chain cleavage, resulting in the degradation of lignin, cellulose, and hemicellulose within the *S.psammophila* sand barriers. This outcome is consistent with previous findings on aging in wood materials (Temiz et al., 2006; Wang et al., 2009a).

The intensity of the absorption peak of the chemical functional groups of lignin was within the range of 800-1800 cm^-1^ wave number, exhibited significant expansion and vibration of *S.psammophila* sand barriers during accelerated aging induced by ultraviolet irradiation and water. The majority of the organic matter in wood materials undergoes degradation when exposed to sunlight, water, oxygen, and other environmental factors (Fu, 2009). The ultraviolet irradiation not only induces the fragmentation, cross-linking, subsequent generation of organic oxides in the organic substrate, leading to photodegradation as observed in previous studies (Rowell, 2005, 1984). The rapid decomposition of lignin is attributed to the enhanced hydrolytic action of water, resulting in the cleavage of lignin macromolecules. After accelerated aging for 576 hours, the cellulose crystallinity of *S.psammophila* sand barriers decreased by 14.62%, indicating that ultraviolet irradiation and water can accelerate the degradation process of lignin, cellulose, and hemicellulose within the *S.psammophila* sand barriers, thereby disrupting the integrity of the cell wall and accelerating the progress of damage. Prolonged exposure to light and environmental humidity (moisture) leads to the degradation of lignin in wood components, resulting in a conversion of a portion of the cellulose crystal region into an amorphous state and subsequently causing a decrease in relative crystallinity (Li et al., 2019).

The XPS analysis results revealed that the oxidation states of C2 and C3 in the sand barrier increased by 40.16% and 12.24%, respectively, under the combined influence of ultraviolet irradiation and water. In the hydroxyl (O-C-O), carbonyl (C=O), and carboxyl (O-C=O) configurations, element C exhibits a higher electron binding energy. Consequently, the chemical bonds on the surface undergo oxidation reactions that tend to be more stable, resulting in enhanced bond stability (Wang et al., 2019; Wang et al., 2009b). The O/C ratio displayed an ascending trend, indicating the rapid degradation of lignin in the *S.psammophila* sand barriers under the combined effect of ultraviolet irradiation and water, resulting in a relative decrease in lignin content. This study indirectly indicates that lignin is less stable than polysaccharides when exposed to ultraviolet irradiation.

The crumpling and cracking of the cell wall facilitate the penetration of ultraviolet-irradiated water into both the cell cavity and the cell wall, thereby enhancing the interface between the *S. psammophila* sand barriers structure and the light-water interaction (Wang et al., 2020; Wang et al., 2021b). Upon complete absorption of ultraviolet light, the *S.psammophila* sand barriers undergoes photochemical reactions that result in the generation of free radical species.Subsequently, in the presence of oxygen and water, a series of molecular chain scissions is initiated, ultimately leading to the degradation of the *S.psammophila* sand barrier. The degradation of lignin, cellulose, and hemicellulose within the *S.psammophila* sand barrier significantly affects its stability and durability. Generally, during the ultraviolet irradiation aging treatment process of *S.psammophila* sand barriers, the incorporation of water mist treatment accelerates the degradation process.

It can be deduced that the degradation of the performance of *S.psammophila* sand barriers in the sandy area is attributed to the aging resulting from ultraviolet irradiation and the combined effect of water, which to some extent reduces the protective effect of the *S.psammophila* sand barriers.

## 5 Conclusion

1. Prolonged exposure to sunlight (ultraviolet irradiation) and humidity (water) can lead to the deterioration of the structural integrity and performance of *S.psammophila* sand barriers, particularly the chemical properties of lignin.

2. After 576 hours of accelerated aging, the cracks within the structure deepened and expanded, thereby accelerating the aging and metamorphic process of *S.psammophila* sand barriers.

3. The future process of desertification control requires the implementation of waterproofing measures and protection against ultraviolet irradiation to enhance the longevity of the *S.psammophila* sand barrier.

## Data Availability

The original contributions presented in the study are included in the article/supplementary material. Further inquiries can be directed to the corresponding author.
